# Study on the driving mechanism of lagged effects based on different time scales in a karst drainage basin in South China

**DOI:** 10.1038/s41598-023-36098-0

**Published:** 2023-06-08

**Authors:** Zhonghua He, Shan Pan, Xiaolin Gu, Mingjin Xu, Maoqiang Wang

**Affiliations:** 1 School of Geography and Environmental Science, Guizhou Normal University, Guiyang, 550001 Guizhou China; 2 School of Geography and Environmental Science, Guizhou Normal University/National Engineering Technology Institute for Karst, Guiyang, Guizhou China; 3 Guizhou Hydrology and Water Resources Bureau, Guiyang, 550002 Guizhou China

**Keywords:** Environmental sciences, Hydrology

## Abstract

Compared to earthquakes and volcanoes, drought is one of the most damaging natural disasters and is mainly affected by rainfall losses, especially by the runoff regulation ability of the underlying watershed surface. Based on monthly rainfall runoff data recorded from 1980 to 2020, in this study, the distributed lag regression model is used to simulate the rainfall-runoff process in the karst distribution region of South China, and a time series of watershed lagged-flow volumes is calculated. The watershed lagged effect is analyzed by four distribution models, and the joint probability between the lagged intensity and frequency is simulated by the copula function family. The results show that (1) the watershed lagged effects simulated by the normal, log-normal, P-III and log-logistic distribution models in the karst drainage basin are particularly significant, with small mean square errors (*MSE*s) and significant time-scale characteristics. (2) Affected by spatiotemporal distribution differences in rainfall and the impacts of different basin media and structures, the lag response of runoff to rainfall differs significantly among different time scales. Especially at the 1-, 3- and 12-month scales, the coefficient of variation (*C*_*v*_) of the watershed lagged intensity is greater than 1, while it is less than 1 at the 6- and 9-month scales. (3) The lagged frequencies simulated by the log-normal, P-III and log-logistic distribution models are relatively high (with medium, medium–high and high frequencies, respectively), while that simulated by the normal distribution is relatively low (medium–low and low frequencies). (4) There is a significant negative correlation (*R* < − 0.8, *Sig*. < 0.01) between the watershed lagged intensity and frequency. For the joint probability simulation, the fitting effect of the gumbel Copula is the best, followed by the Clayton and Frank-1 copulas, and while that of the Frank-2 copula is relatively weak. Consequently, the propagation mechanism from meteorological drought to agricultural or hydrological drought and the conversion mechanism between agricultural and hydrological drought are effectively revealed in this study, thereby providing a scientific basis for the rational utilization of water resources and drought resistance and disaster relief in karst areas.

## Introduction

Most of the rainfall, before entering a river network, is stored in the hydrological subsystem, such as in ice and snow, soil water, groundwater and reservoirs, resulting in runoff exhibited a temporal lag compared to rainfall^1^; this lagged intensity or frequency is greatly affected by the rainfall time, watershed water storage capacity and human activities^2–4^. While the watershed lagged effect has rarely attracted the attention of scholars, it is very important for studies on drought mechanisms, especially those on drought propagation or conversion mechanisms between different types of droughts^5–8^. The lag response of runoff to rainfall in karst basins is 3 months^9^. The propagation time (PT) is about 1 month from meteorological drought to hydrological drought, and 2 months from hydrological to agricultural drought. The PT from a meteorological drought to agricultural drought is 1–2 months in summer and 2–7 months in spring, while it is 2–3 months from a hydrological drought to a vegetation drought^8,10–14^. These PTs may be the result of the response time of runoff to climate change depending on the climatic conditions of the basins, the media characteristics of catchment areas and the impacts of human activities^15,16^. For instance, climate change conditions, such as the rainfall intensity, temperature and wind speed, result in variable infiltration rates and peak runoff, further influencing the amount of runoff from bare land^17^. Vertically layered vegetation has a cutting effect on the rainfall velocity and reduces the shock and destruction of raindrops to the watershed surface^18^; the horizontal distribution of vegetation delays the lateral velocity of rainfall on the watershed surface and increases the drainage infiltration rate. Some precipitation may directly reach the surface soil and cause certain damage to the ground surface when the vegetation cover is less than 100%^19^. Therefore, watershed vegetation has a strong controlling effect on rainfall-runoff processes^20^. The geomorphic types and spatial distributions also significantly impact the secondary distribution of rainfall on the watershed surface and underground, especially in terms of the flood peak and low-flow runoff modulus^19^. Watershed soil is an important component of basin media^21^, and the “pore space and soil thickness in the soil determine the water storage capacity in the hillslope, and its spatial distribution pattern can affect the response relationship between rainfall and runoff”^22,23^. “Soil structure is a direct controlling factor affecting infiltration capacity, and is especially affected by plant roots, and even dynamic changes during the growing season”^24^. The watershed lithology is an important factor affecting the rainfall-runoff process in small and medium-sized basins. For instance, the higher the proportion of limestone is, the smaller the low-flow runoff modulus is, and the higher the proportion of dolomite is, the larger the low-flow runoff modulus is. The higher the proportion of peak cluster depression is, the smaller the low-flow runoff modulus is, and the higher the proportion of peak forest karst (basin) is, the larger the low-flow runoff modulus is^25^. Consequently, the impacts of the watershed media and spatial combination on the rainfall-runoff process are extremely significant and influence the watershed water storage capacity^21^. At the same time, the impact of large-scale atmospheric circulation on rainfall runoff processes cannot be ignored. Some previous studies have shown that precipitation extremes in China are strongly affected by several large-scale climate variability modes, including the El Niño–Southern Oscillation (ENSO), Pacific Decadal Oscillation (PDO), Indian Ocean Dipole (IOD), and Atlantic Multidecadal Oscillation (AMO). Previous studies have documented a well-established relationship between seasonal rainfall in China and the positive or negative phase of ENSO^26–29^.

As mentioned above, runoff and rainfall do not occur at the same time, and these factors have linear relationships in areas with relatively thin watershed thicknesses and nonlinear relationships in areas with relatively thick watershed thicknesses^22^. At present, the correlation between rainfall and runoff has been analyzed mainly by employing Pearson correlation analysis (PCC) or continuous wavelet transform (CWT). The maximum PCC (MPCC) or cross wavelet transform method is used to represent the propagation time (PT) of the rainfall-runoff process^5,30^ and to discuss the impacts of mainly large-scale atmospheric circulation and local climate change on rainfall-runoff processes. However, there are few studies on the impacts of rainfall at different scales, underlying surface media, and spatial structures on watershed lagged effects^31^. Therefore, the objectives of this study are (i) to use a distributed lag model to simulate rainfall-runoff processes in karst basins and calculate the total amount of watershed lagged flow; (ii) to fit the time series of the lagged-flow volume by the normal distribution, log-normal distribution, P-III distribution and log-logistic distribution and choose the optimal model; (iii) to analyze the characteristics of the watershed intensity and frequency and reveal the mechanism of lagged effects in karst basins; and (iv) to use Copula functions to discuss the joint probability between the watershed lagged intensity and frequency and to reveal the spatial distribution and temporal evolution of lagged effects in karst basins. Therefore, this study systematically discusses the lag effect mechanism based on watershed water storage in karst drainage basins to lay a theoretical basis for effectively revealing propagation from meteorological drought to agricultural drought or hydrological drought and provide technical guidance for drought relief in karst areas.

## Study area

**Figure 1:**
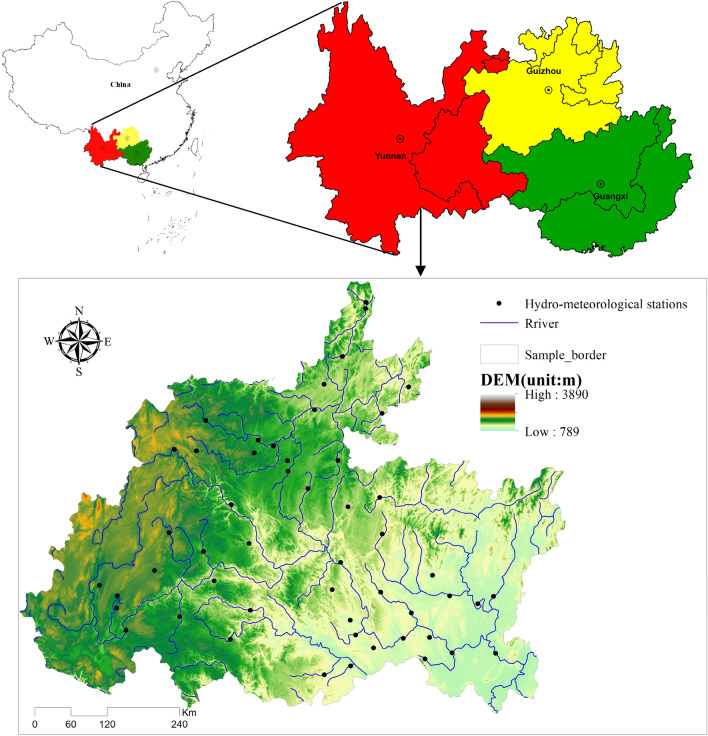
Spatial distribution map of hydrometeorological stations in the research areas. Map was created with ArcGIS version 10.3 (https://www.arcgis.com/).

South China, centered on Guizhou, Yunnan and Guangxi, is a typical karst distribution area containing cone karst, swamp karst and tower karst regions. The sample areas controlled by the 51 hydrometeorological stations in South China selected in this paper are shown in Fig. 1; these areas are enclosed by the northern latitudes of 22°42′57″–29°13′11″ and eastern longitudes of 101°55′55″–110°55′45″ with an average elevation of 1065.62 m. Together, these regions comprise most areas of Guizhou Province (37.97%), the southeast part of Yunnan Province (25.36%) and the northwestern and northern regions of Guangxi Province (36.67%), with an area 352,526 km^2^. The study area is located in the humid climate area of the northern subtropical zone and the semihumid climate area of the southern subtropical zone. Annual rainfall is abundant but has an uneven spatiotemporal distribution. The average annual precipitation in the whole region is 1000–1300 mm, and the mean annual temperature is 16–23 °C. The study area is bounded by the Wumeng–Miaoling Mountains and belongs to the Yangtze River Basin (YRB) and the Pearl River Basin (PRB). That is, the northern region contains the Jinsha River system, Wujiang River system and Dongting Lake system in the YRB, whereas the southern region comprises the Nanpan River system, Beipan River system, Hongshui River system, Duliu River system (in Guizhou), Yuanjiang River system (in Yunnan), and Xijiang River system (in Guangxi) in the PRB. The land use types mainly include forestlands (60.2%), cultivated lands (20.7%), grasslands (16.1%), construction lands (2%), and water areas (0.98%).

## Data and methods

### Research data

The meteorological data used in this paper were obtained mainly from the Global Land Data Assimilation Systems (GLDAS) website (https://ldas.gsfc.nasa.gov/) at a spatial resolution of 0.25° × 0.25°, as well as from the China Meteorological Data Sharing Service System (http://data.cma.cn/site/index.html). Hydrological data provided by the Ministry of Water Resources of the People’s Republic of China (*Hydrologic Year Book of People’s Republic of China Hydrologic Data of Yangtzi River Basin, Volume 6 and Hydrologic Data of Pearl River Basin, Volume 8*) were analyzed for their representativeness, reliability and homogeneity. Fifty-one hydrometeorological stations (including 11 in Yunnan, 19 in Guangxi, and 21 in Guizhou; Fig. 1) were chosen, and monthly rainfall-runoff data from 1980 to 2020 were calculated. By combining a comprehensive hydrogeological map, the watershed elevation and water system characteristics were automatically extracted based on a DEM with a spatial resolution of 30 m by ArcGIS 10.3. To eliminate the influence of different catchment areas, the data used in this study were standardized.

### Research methods

#### Simulation of the rainfall-runoff process

- (1) Calculation of lagged-flow volume

First, the watershed lagged effect was analyzed and judged using the cross y, x of Eviews 9.0.

Second, a distributed lag regression model was built by employing the PDL of Eviews 9.0, namely,

1 $$ {\text{LS y }}\alpha {\text{ PDL}}\left( {{\text{x}},\;{\text{k}},\;{\text{m}},\;{\text{d}}} \right) $$

The distributed lag regression model was expressed as follows:

2 $$ {\text{y}}_{t} = \alpha + \sum\limits_{i = 0}^{k} {\beta_{t - i} x_{t - i} + \mu_{t} } $$

where ${\text{y}}_{t}$ refers to the runoff volume (theoretical value) at the *tth* time scale and $\beta_{t - i}$ and $x_{t - i}$ are the lagged coefficient and variable (rainfall volume) at the *tth* time scale and *ith* lagged period, respectively. $\beta_{t - i} x_{t - i}$ is the runoff volume (${\text{y}}_{t}$) at the *tth* time scale generated by the rainfall volume at the *ith* lagged period; the larger the value of $\beta_{t - i} x_{t - i}$ is, the larger the contribution rate of the *ith* lagged variable ($x_{t - i}$) to the runoff volume (${\text{y}}_{t}$) is. $\mu_{t}$ is a random variable ($\mu$) in the *tth* time scale, $\alpha$ is a constant, k is the length of the lagged period, m is a polynomial order, and d is a controlling parameter of lagged characteristics. In this paper, k = 3, m = 2, and d is the defect value^9^.

Finally, the watershed lagged effect is reflected by the difference (*D*) between the actual observed value and the theoretical runoff volume value. Namely, the negative difference (*D*) demonstrates that the basin has the characteristics of lagged effects, and the larger the negative value *D* is, the stronger the watershed lagged effect is^9^.

- (2) Analysis of watershed lagged effect characteristics

In univariate analysis, seven marginal distributions are commonly used to simulate the runoff process^32^. Based on comprehensive consideration, four distributions, mainly including the normal distribution, log-normal distribution, P-III distribution and log-logistic distribution, were selected in this paper to analyze the time series of the lagged-flow volume^33,34^. The model parameters were estimated mainly by moment estimation, and the fitting result of each model was tested by the Kolmogorov‒Smirnov test (K-S) and mean square error (*MSE*).

- (3) Calculation of the watershed lagged index (*LI*)

The lagged index (*LI*) can be calculated as follows:

3 $$ LI_{{{\text{t}},m}} = \frac{{D_{t,m} - \overline{D}_{{\text{t,m}}} }}{{S_{t,m} }};\;t = 1,\;3,\;6,\;9,\;12,\;m = 1,\;2,\;3,\;4 $$

where $D_{{{\text{t}},m}} = y_{{{\text{actual observed value }}(t)}} - y_{{{\text{theoretical value }}(t)}}$ refers to the lagged-flow volume of the *mth* model in the *tth* time scale and $\overline{D}_{{\text{t,m}}}$ and ${\text{S}}_{{\text{t,m}}}$ refer to the mean value and standard deviation of the lagged-flow volume in the *tth* time scale, respectively.

When *LI* is positive, it indicates a normal (i.e., nonlagged) effect. When the *LI* is negative, the larger the absolute value is, the more serious the lagged intensity is. According to the lagged index (*LI*) and frequency (*F*), the watershed lagged intensity and frequency can be divided into five levels (Table 1)^9,35,36^.

**Table 1:** The grade standards of the watershed lagged intensity and frequency.

Lagged intensity		Lagged frequency	
Level	Index (*LI*)	Level	Index (*F*)
Nonlagged effect	0.0 ≤ *LI*	Low frequency	0–20%
Mild lagged effect	− 1.0 ≤ *LI* < 0.0	Medium–low frequency	20–40%
Moderate lagged effect	− 1.5 ≤ *LI* < − 1.0	Medium frequency	40–60%
Severe lagged effect	− 2.0 ≤ *LI* < − 1.5	Medium–high frequency	60–80%
Extreme lagged effect	*LI* < − 2.0	High frequency	80–100%

#### Joint probability between the lagged intensity and frequency

- (1) Construction of the copula joint probability

To better reflect the characteristics of the watershed lagged intensity and frequency, two-dimensional Archimedean copulas with simple structures and strong adaptability, such as the Gumbel copula, Clayton and Frank copulas (Table 2), were selected in this study to construct the joint probability between the lagged intensity and frequency in karst drainage basins in South China^6,33,34^.

**Table 2:** Copula function family between the watershed lagged intensity and frequency.

Copula	Functional form	Relationship between $\theta$ and $\tau$
Gumbel copula	$$F\left( {p,z} \right) = C\left( {u,v} \right) = \exp \left\{ { - \left[ {\left( { - \ln u} \right)^{\theta } + \left( { - \ln v} \right)^{\theta } } \right]^{{{\raise0.7ex\hbox{$1$} \!\mathord{\left/ {\vphantom {1 \theta }}\right.\kern-0pt} \!\lower0.7ex\hbox{$\theta $}}}} } \right\}$$	$$\tau = 1 - \frac{1}{\theta },\theta \in [1,\infty )$$
Clayton copula	$$F\left( {p,z} \right) = C\left( {u,v} \right) = \left( {u^{ - \theta } + v^{ - \theta } - 1} \right)^{{{\raise0.7ex\hbox{${ - 1}$} \!\mathord{\left/ {\vphantom {{ - 1} \theta }}\right.\kern-0pt} \!\lower0.7ex\hbox{$\theta $}}}}$$	$$\tau = \frac{\theta }{2 + \theta },\theta \in (0,\infty )$$
Frank copula	$F\left( {p,z} \right) = C\left( {u,v} \right) = - \frac{1}{\theta }\ln \left[ {1 + \frac{{\left( {\ell^{ - \theta u} - 1} \right)\left( {\ell^{ - \theta v} - 1} \right)}}{{\left( {\ell^{ - \theta } - 1} \right)}}} \right]$	$\tau = 1 + \frac{4}{\theta }\left[ {\frac{1}{\theta }\int_{0}^{\theta } {\frac{t}{{\ell^{t} - 1}}dt - 1} } \right],\theta \in R$

Among them, the parameter $\theta$ and Kendall’s correlation coefficient $\tau$ shown in Table 2 can be calculated by the following formulas:

4 $$ \tau = \left( {C_{n}^{2} } \right)^{ - 1} \sum\limits_{i < j} {sign\left[ {\left( {x_{i} - x_{j} } \right)\left( {y_{i} - y_{j} } \right)} \right]} $$

5 $$ sign[(x_{i} - x_{j} )(y_{i} - y_{j} )] = \left\{ \begin{gathered} 1,(x_{i} - x_{j} )(y_{i} - y_{j} ) > 0 \hfill \\ 0,(x_{i} - x_{j} )(y_{i} - y_{j} ) = 0 \hfill \\ - 1,(x_{i} - x_{j} )(y_{i} - y_{j} ) < 0 \hfill \\ \end{gathered} \right. $$

where $C_{n}^{2}$ represents the number of combinations and* x*_*i*_ and *y*_*i*_ represent the time series of the lagged intensity and frequency, respectively. The larger the Kendall’s correlation coefficient $\tau$ is, the more significant the correlation between variables is.

- (2) Optimal test of the copula joint probability

The copula functions (Table 2) were selected to fit the joint probability between the lagged intensity and frequency, and it was necessary to use the goodness of fit test index to optimize and determine the best fit among these functions. Typically used goodness of fit test indicators include the mean square error (*MSE*), Akaike information criterion (*AIC*) and Bayesian information criterion (*BIC*); these indicators are calculated using the following formulas^37–39^:

6 $$ MSE = \sqrt {\frac{1}{n}\sum\limits_{i = 1}^{n} {\left( {p - p_{i} } \right)^{2} } } $$

7 $$ AIC = n\ln (MSE) + 2m $$

8 $$ BIC = n\ln (MSE) + m{\text{ln(}}n{)} $$

where $p$ and $p_{i}$ represent the empirical and theoretical frequencies and *m* and *n* are the number of model parameters and sample observations, respectively. The smaller the *MSE*, *AIC* and *BIC* values are, the better the model fitting effect is.

## Results and analysis

### Goodness-of-fit test of the lagged-flow volume distributions at different time scales

**Figure 2:**
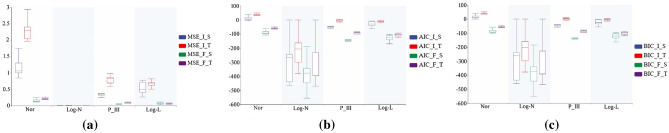
MSE (**a**), *AIC* (**b**) and *BIC* (**c**) results of the normal, log-normal, P-III and log-logistic distributions.

To reveal the characteristics of watershed lagged effects, this paper simulated the rainfall-runoff process according to Formulas (1) and (2) and calculated the total amount of watershed lagged flow^9^. The time series of the lagged-flow volume were fitted using four functions, the normal distribution, log-normal distribution, P-III distribution and log-logistic distribution models, based on different time scales (1, 3, 6, 9 and 12 months). The lagged index (*LI*) was computed based on Formula (3), and the fitting effects of the lagged intensity and frequency were discussed by employing the *MSE*, *AIC* and *BIC* values, as shown in Fig. 2. This study found that the *MSEs* of the watershed lagged effects simulated by the four models were relatively small (Fig. 2a). Our results agree with^40^, who reported a linear relationship between rainfall and runoff in the Pearl River Basin (PRB); in particular, the log-normal distribution was the most suitable probability distribution for fitting the streamflow at the yearly or 6-month period^41^. The *AIC* and *BIC* values of the log-normal distribution simulation were maximally negative, especially at the 1–12 month scales, with relatively large differences, and those of the other distributions were also relatively large but with smaller differences (Fig. 2b,c). These results indicate that the log-normal distribution is the best-fitting distribution for the watershed lagged effects on significant time scales, and the fitting effects of the other distributions were relatively weak^34,42^.

**Figure 3:**
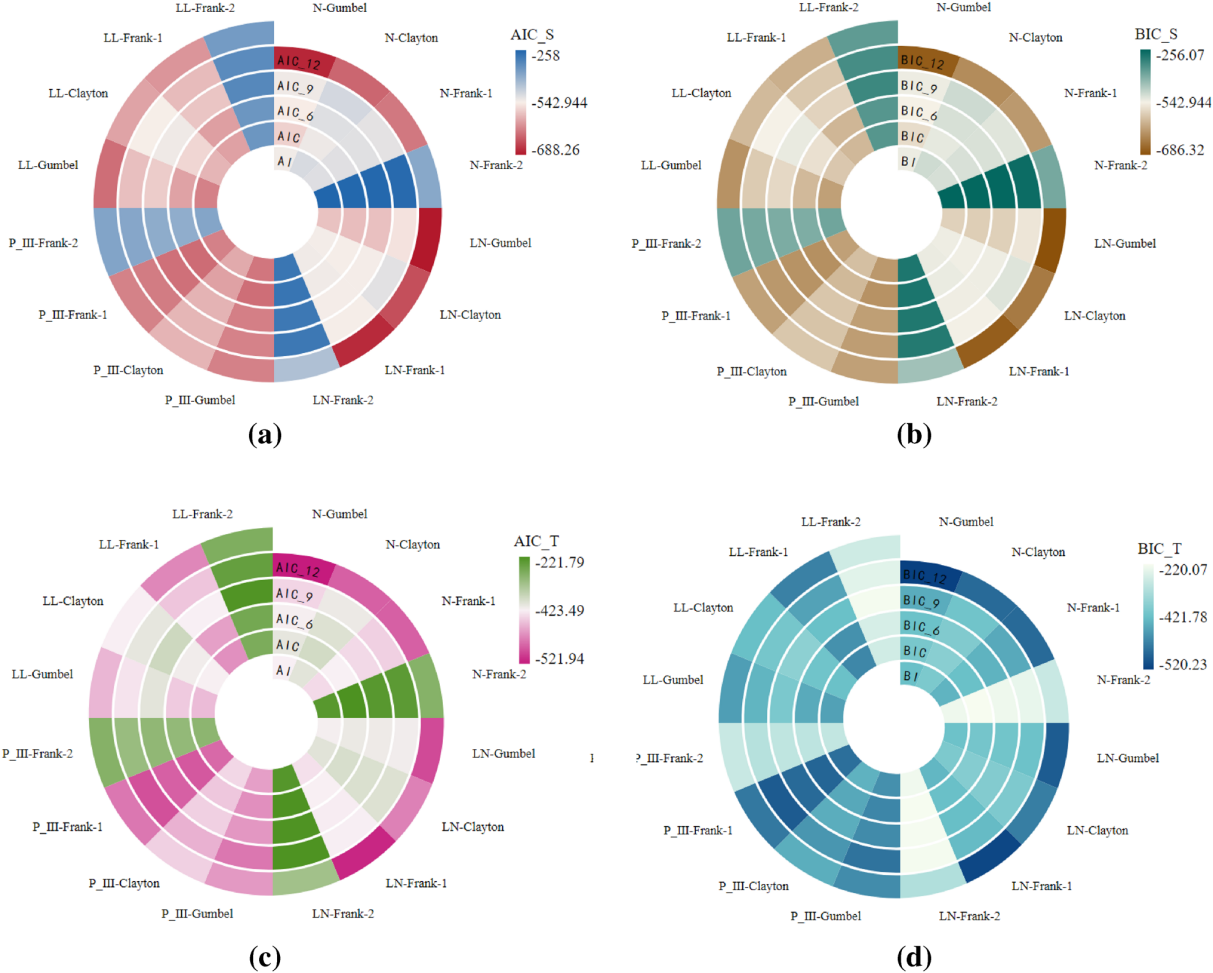
*AIC* and *BIC* results of the joint probability between the lagged intensity and frequency in terms of different time scales.

Similarly, to discuss the relationship between the watershed intensity and frequency, the joint probability was calculated using the copula function in this study (Table 2). According to the relationship between $\theta$ and $\tau$, the parameter $\theta$ of the Frank copula had two values, $\theta$ < 1 (Frank-1 copula) and $\theta$ > 1 (Frank-2 copula), and the fitting effects of the copula function were tested by the *AIC* and *BIC* (Fig. 3a–d). The Gumbel's *AIC* and *BIC* values, whether from the perspective of the spatial distribution or time change considered in this study, were maximally negative; that is, the fitting effect of the Gumbel copula was the best, followed by the Clayton and Frank-1 copulas, while the Frank-2 copula was relatively weak. These results are similar to the study of Jha et al.^43^, who reported that the Gumbel copula was the most suitable copula for representing the joint distribution of the drought duration and severity.

### Spatiotemporal evolution characteristics of lagged intensity in the karst drainage basin

**Figure 4:**
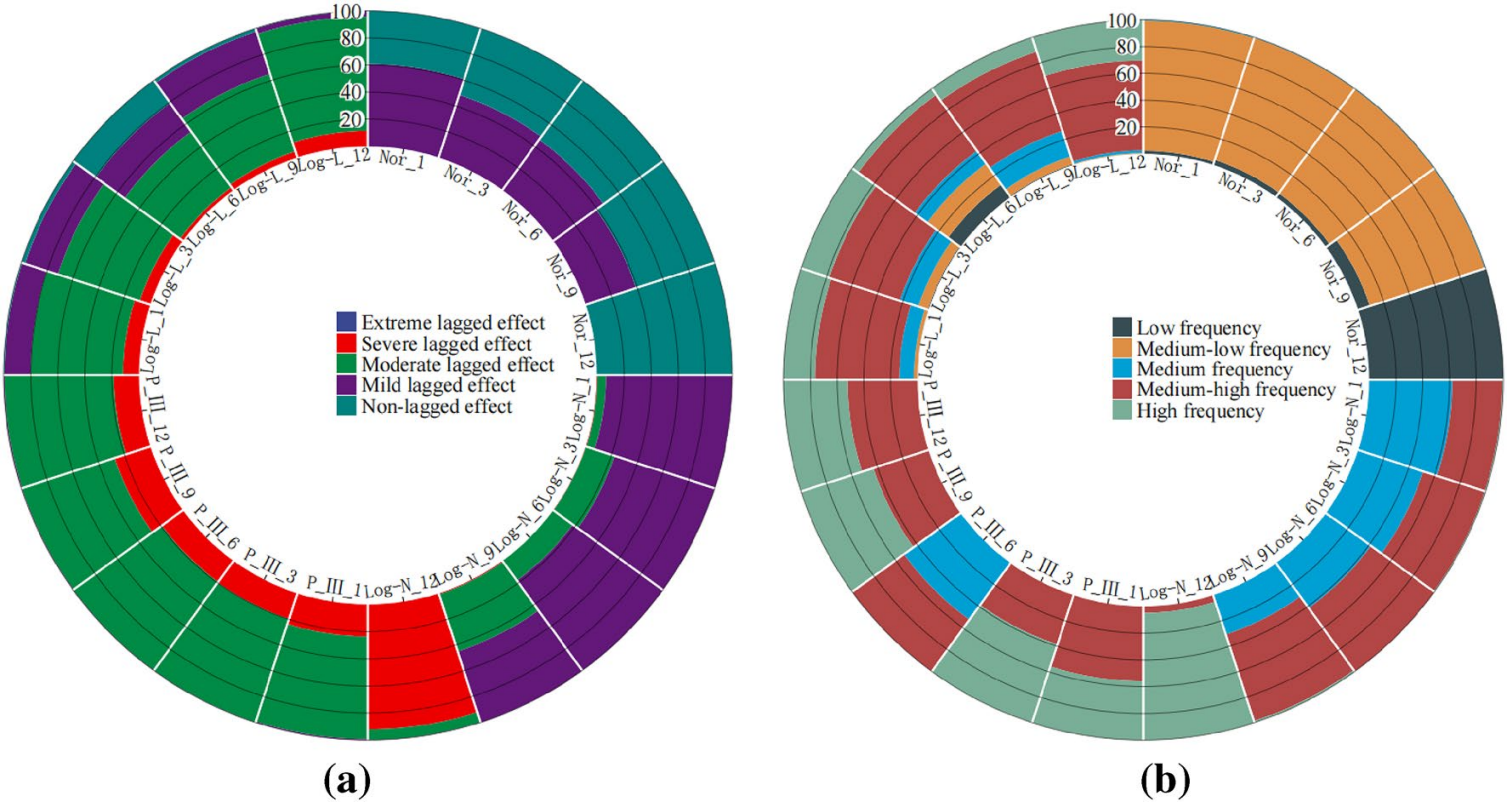
The areal percentages of the watershed lagged intensity (**a**) and frequency (**b**) at different time scales.

**Figure 5:**
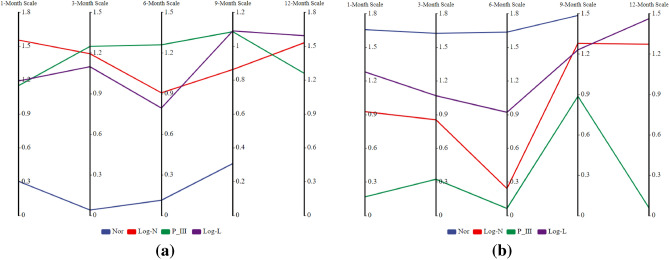
The spatial variability (*C*_*v*_) in the watershed lagged intensity (**a**) and frequency (**b**) simulated by different distributions at different time scales.

**Figure 6:**
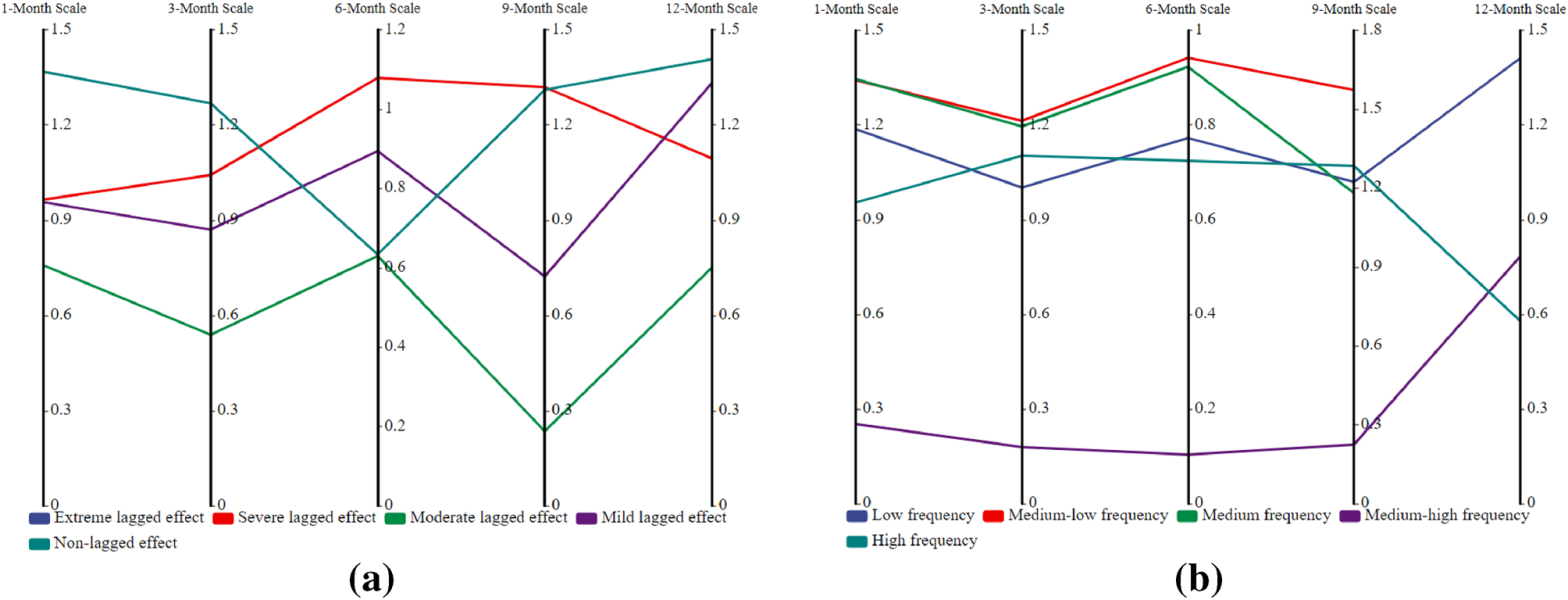
The spatial variability (*C*_*v*_) in the different grades of watershed lagged intensity (**a**) and frequency (**b**) at different time scales.

**Figure 7:**
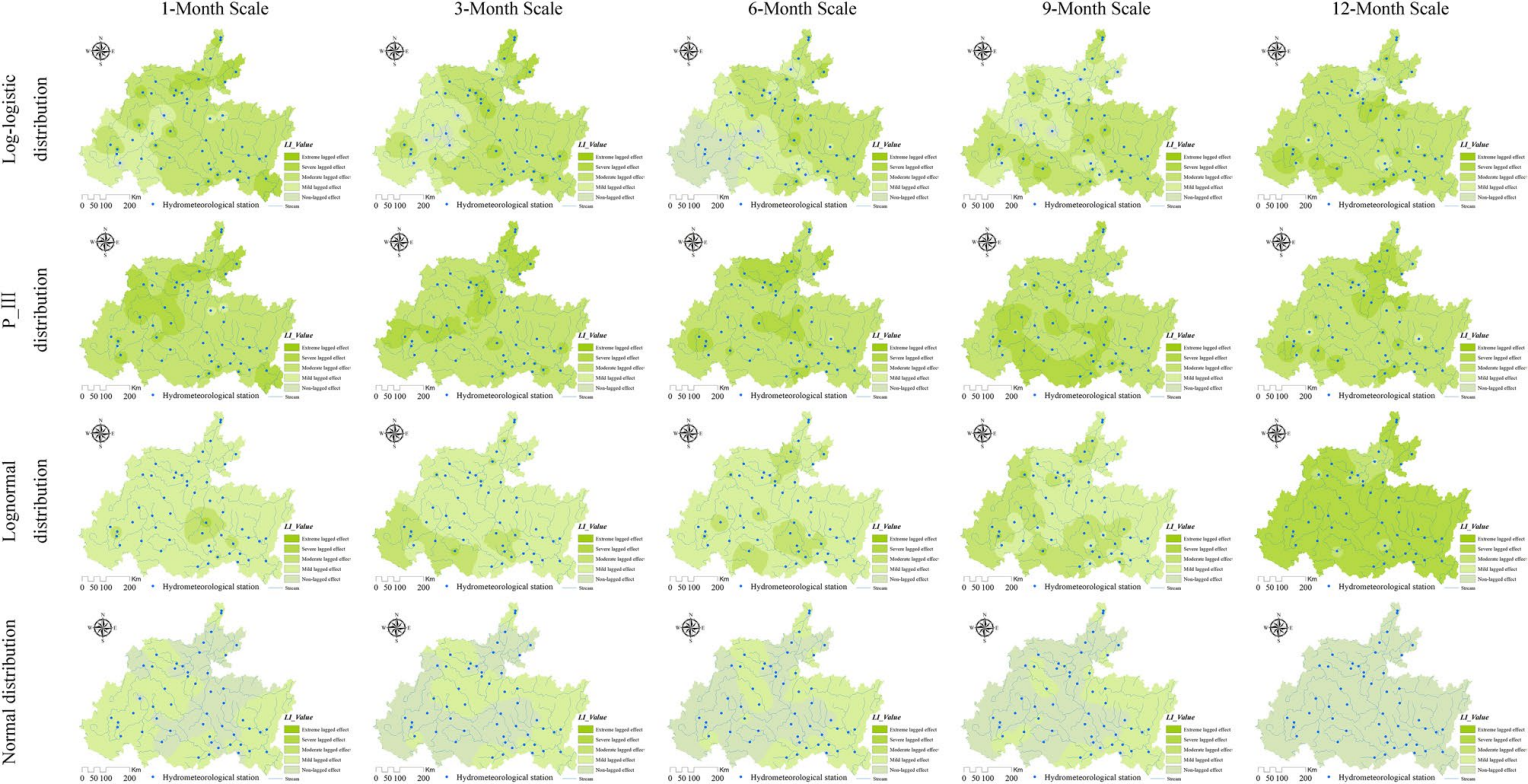
Spatial distribution characteristics of the watershed lagged intensity at different time scales. Map was created with ArcGIS version 10.3 (https://www.arcgis.com/).

According to the smallest criterion of the mean square error (*MSE*) in this study, the watershed lagged intensity was discussed using the normal, log-normal, P-III and log-logistic distributions in terms of different time scales. The fitting effects of these four distributions varied greatly, and the lagged intensity mainly presented a spatial pattern of strong in the east and south and weak in the west and north (Fig. 7). Among the distributions, mild to severe lagged levels were found in the log-normal and P-III distribution results, nonlagged to severely lagged levels were observed in the log-logistic distribution results, and nonlagged and mild lagged levels were shown in the normal distribution results (Fig. 7). It is likely that the watershed lagged effect was deeply affected not only by the spatial distribution of rainfall but also by the response of the watershed media structure to climate change^44^. However, on significant time scales, the watershed lagged effect is caused by seasonal variations in rainfall^45,46^. This study found that the distribution area of the severely lagged level as simulated by the log-normal distribution on 12-month scale was 92.12% of total area, while those of the mildly lagged level on the 1- and 6-month scales were 93.09% and 82.04%, respectively (Fig. 4a); the distribution areas of the moderately lagged level simulated by the P-III and log-logistic distributions were 72.07%-80.69% and 50.65%-84.36%, respectively; and the distribution area of the mildly lagged level simulated by the normal distribution on 1–12-month scales gradually decreased while that of the nonlagged level gradually increased (Fig. 4a). Moreover, the time scale also has an impact on the model fit. For example, the coefficients of variation (*C*_*v*_) of the watershed lagged intensities at the 1–12-month scales were greater than 0.8 (except that of normal distribution, for which *C*_*v*_ < 0.3), especially at the 1-, 3- and 12-month scales, when they were greater than 1 (*C*_*v*_ > 1) (Fig. 5a). The *C*_*v*_ values of the nonlagged (except at the 6-month scale) and severely lagged (except at the 1-month scale) levels were greater than 1, and those of the mild (except at the 12-month scale) and moderately lagged levels were less than 1 (Fig. 6a). This may have been because the average annual rainfall at short time scales (1 and 3 months) was larger with smaller changes, and the runoff regulation of karst basins is relatively weak; conversely, the average annual rainfall at long time scales (12 months) is smaller with larger changes, and the runoff regulation effect of karst basins is relatively strong, resulting in a significant difference in the lag response of runoff to rainfall among different time scales^5^. The variations in the annual rainfall and runoff regulation of karst basins at 6 and 9 months (medium time scales) are greater than those at 1 and 3 months and less than those at 12 months, leading to a smaller difference in the lag response of runoff to rainfall^47–49^. In addition, watershed lagged effects are deeply affected by the basin media disturbance caused by human activities^4^. From an interdecadal perspective, the watershed lagged effect simulated by the P-III distribution was the most significant and showed moderate and above-moderate levels. The watershed lagged effect simulated by the normal distribution was relatively weak and presented mildly lagged and nonlagged levels (Fig. 9a). The lagged intensity simulated by the log-logistic distribution gradually strengthened from the medium time scales (6 and 9 months) to the short time scales (1 and 3 months) and long time scales (12 months) (Figs. 7 and 9a).

### Spatiotemporal evolution characteristics of the lagged frequency in the karst drainage basin

**Figure 8:**
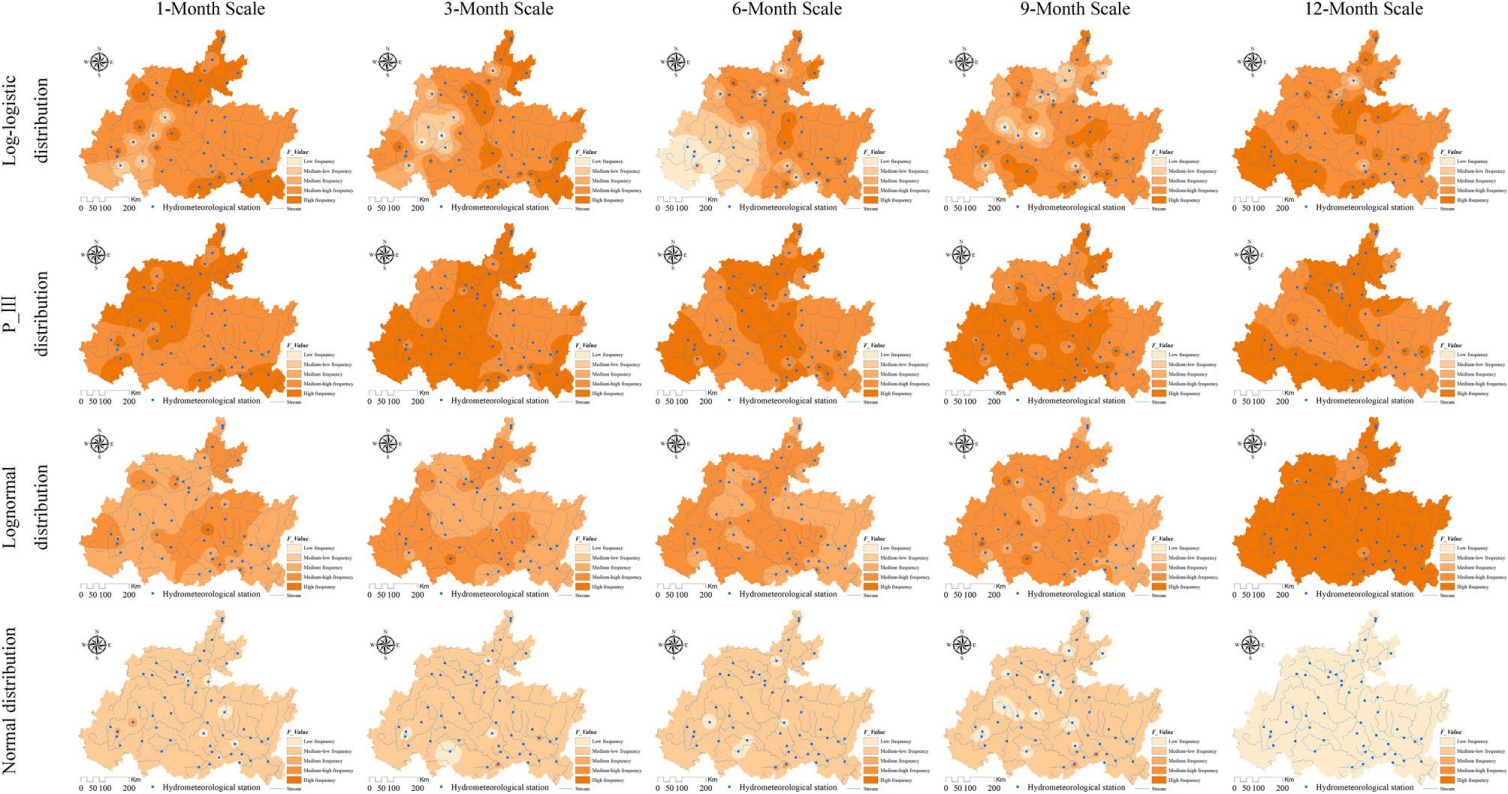
Spatial distribution characteristics of the watershed lagged frequency at different time scales. Map was created with ArcGIS version 10.3 (https://www.arcgis.com/).

**Figure 9:**
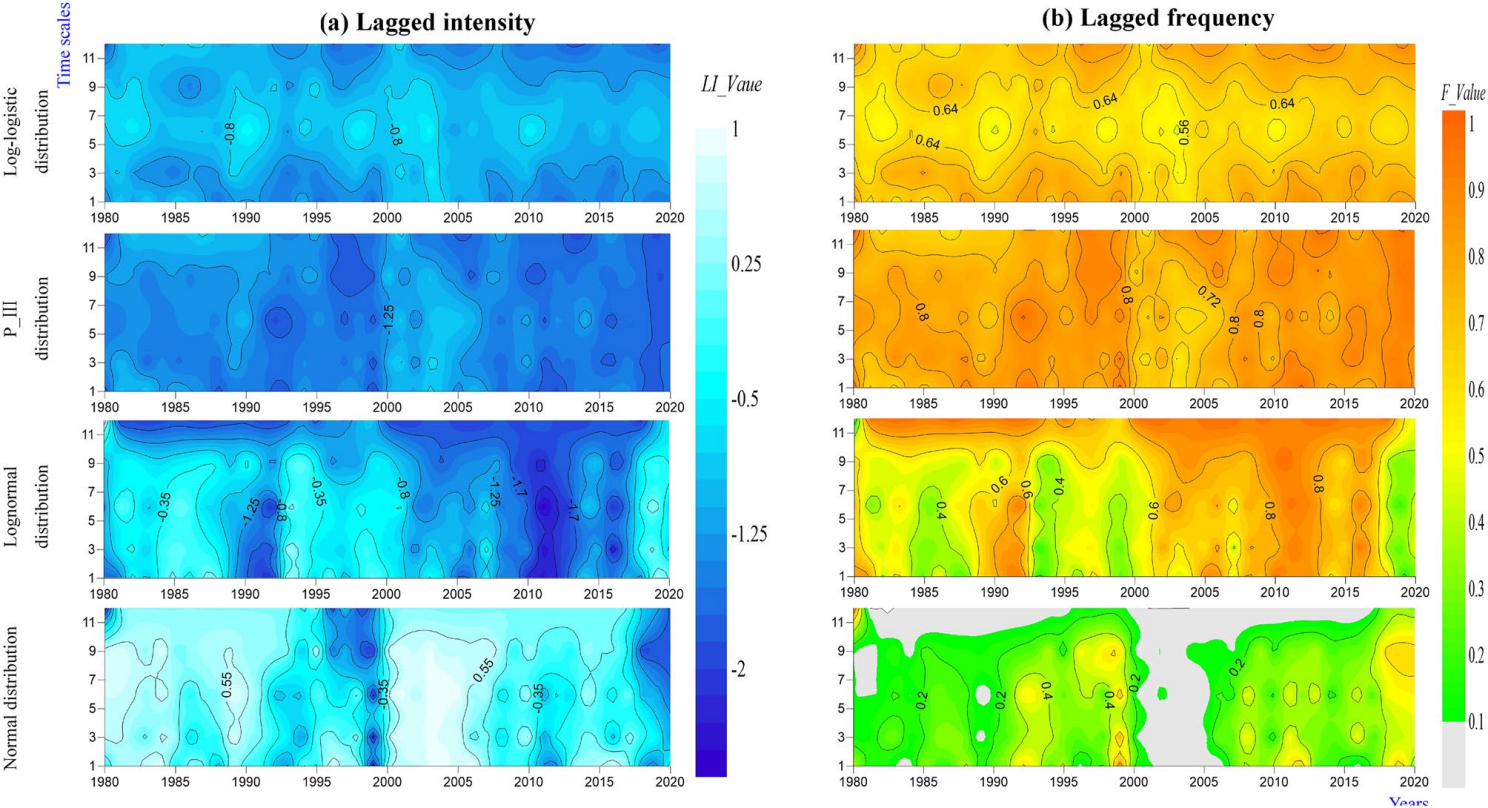
Temporal evolution characteristics of the lagged intensity (**a**) and frequency (**b**) at different time scales.

The watershed lagged intensity is accompanied by a certain lagged frequency. The watershed lagged frequencies simulated by the log-normal, P-III and log-logistic distributions were relatively high, while that in the normal simulation was relatively low (Fig. 8). In particular, medium, medium–high and high frequencies are found in the log-normal and P-III simulation results, and medium–low and low frequencies are shown in the normal simulation results (Fig. 8). These results indicate that the responses of the four distributions to rainfall changes are relatively sensitive and that these distributions can optimally capture the lagged effects of runoff on rainfall^50,51^. Similarly, we also identified an impact of the time scale on the watershed lagged frequency. The distribution area of the high frequency simulated by the log-normal distribution at the 12-month scale reached 95.02% of the total area (Fig. 4b), and that of the moderate-high frequency at the 1–9 month scale gradually increased (37.38–68.02%, Fig. 4b) and showed a decreasing distribution pattern from southwest to northeast (Fig. 8). The lagged frequencies simulated by the P-III distribution at the 6- and 12-month scales presented strong–weak alternating distributions from northwest to southeast (Fig. 8), and the distribution areas of the medium–high frequencies at the 3- and 9-month scales were 61.46% and 56.7%, respectively (Fig. 4b). The lagged frequency simulated by the log-logistic distribution at the 6-month scale increased significantly from southwest to northeast and decreased gradually at the 9- and 12-month scales (Fig. 8). This finding demonstrates that the watershed lagged effects simulated by different models differ significantly with the time-scale characteristics. This may be attributed to the comprehensive impacts of both the time scales of rainfall and spatial differences in the watershed water storage capacity^22,35^. Moreover, the lagged frequency simulated by the normal distribution at different time scales has the largest difference, with *C*_*v*_ > 1.5 (Fig. 5b), followed by the log-logistic simulation, for which *C*_*v,6*_ (0.92) < *C*_*v,3*_ (1.07) < *C*_*v,9*_ (1.23) < *C*_*v,1*_(1.28) < *C*_*v,12*_ (1.46), as well as the log-normal simulation, for which* C*_*v,6*_ (0.24) < *C*_*v,3*_ (0.85) < *C*_*v,1*_ (0.92) < *C*_*v,12*_(1.27) < *C*_*v,9*_ (1.28); the smallest difference was obtained for the P-III simulation with *C*_*v,1–12*_ = 0.05–0.89 (Fig. 5b). For lagged frequencies of different levels at different time scales, the *C*_*v*_ values of the low frequency, moderate-low frequency and medium frequency were all greater than 1 (except at the 6-month scale) and those of the medium–high and high frequencies were less than 0.8 and 0.6–1.3, respectively (Fig. 6b). These results further confirm that the watershed lagged effect was the coupled result of both rainfall changes and runoff regulation, and the influence of time scales cannot be ignored^52,53^. In addition, the impact of age changes on the watershed lagged effect was very significant. Among the studied distributions, the lagged frequencies simulated by the P-III and log-logistic distributions were the highest, followed by that of the log-normal distribution, and the normal distribution simulated the smallest lagged frequency. The lagged frequency simulated by the P-III distribution was greater than 0.6 and reached 0.9 in some years. The lagged frequencies simulated by the log-logistic distribution at different scales represented significant strong–weak alternation within the range of 0.55–0.7 (Fig. 9b). The normal distribution simulation showed significant periodic variations with a strong–weak-strong alternation; namely, 1980–2000 and 2006–2020 were enhancing periods of the lagged frequency (with frequency ranges of 0.1–0.4 and 0.2–0.3, respectively), and 2000–2006 was a weakening period of the lagged frequency (frequency < 0.1) (Fig. 9b). The log-normal distribution simulation displayed two change periods: 1980–2001 was an alternating period of weak-strong–weak frequencies, and 2001–2019 was a strengthening period (Fig. 9b). These results indicate that the influence of human activities on the underlying surface media and structures of the basins affects the runoff generation and confluence mechanisms. These impacts result in significant differences in the response of runoff to rainfall at different time scales or ages^5^.

### Spatiotemporal characteristics of the joint probability between the watershed lagged intensity and frequency

**Figure 10:**
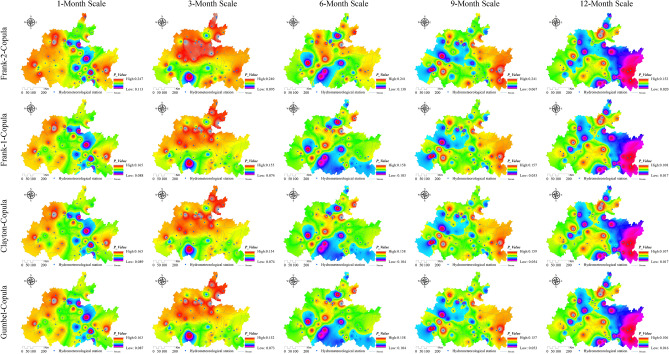
Spatial distribution characteristics of the copula joint probability between the lagged intensity and frequency simulated by the normal distribution. Map was created with ArcGIS version 10.3 (https://www.arcgis.com/).

**Figure 11:**
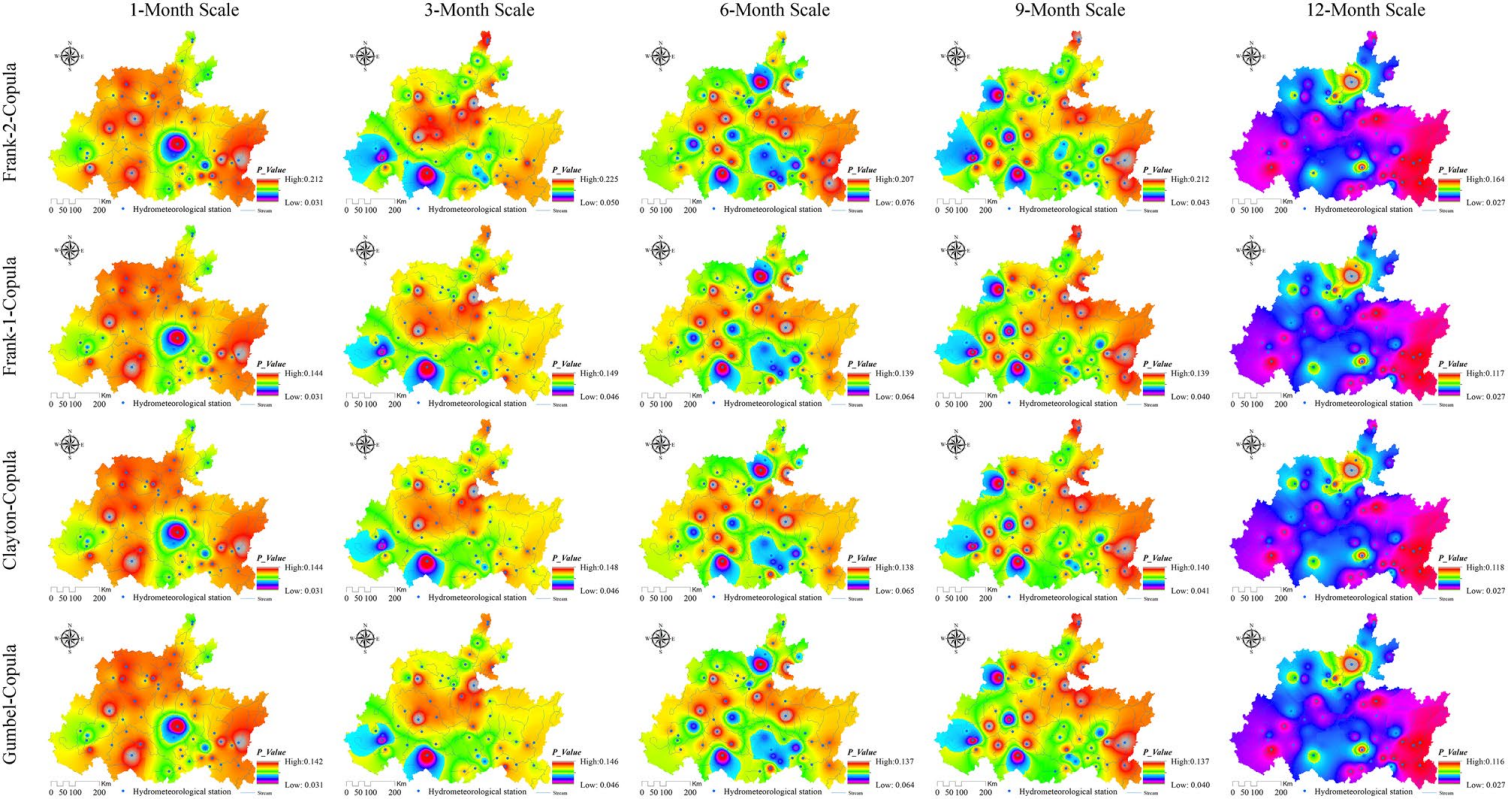
Spatial distribution characteristics of the copula joint probability between the lagged intensity and frequency simulated by the log-normal distribution. Map was created with ArcGIS version 10.3 (https://www.arcgis.com/).

**Figure 12:**
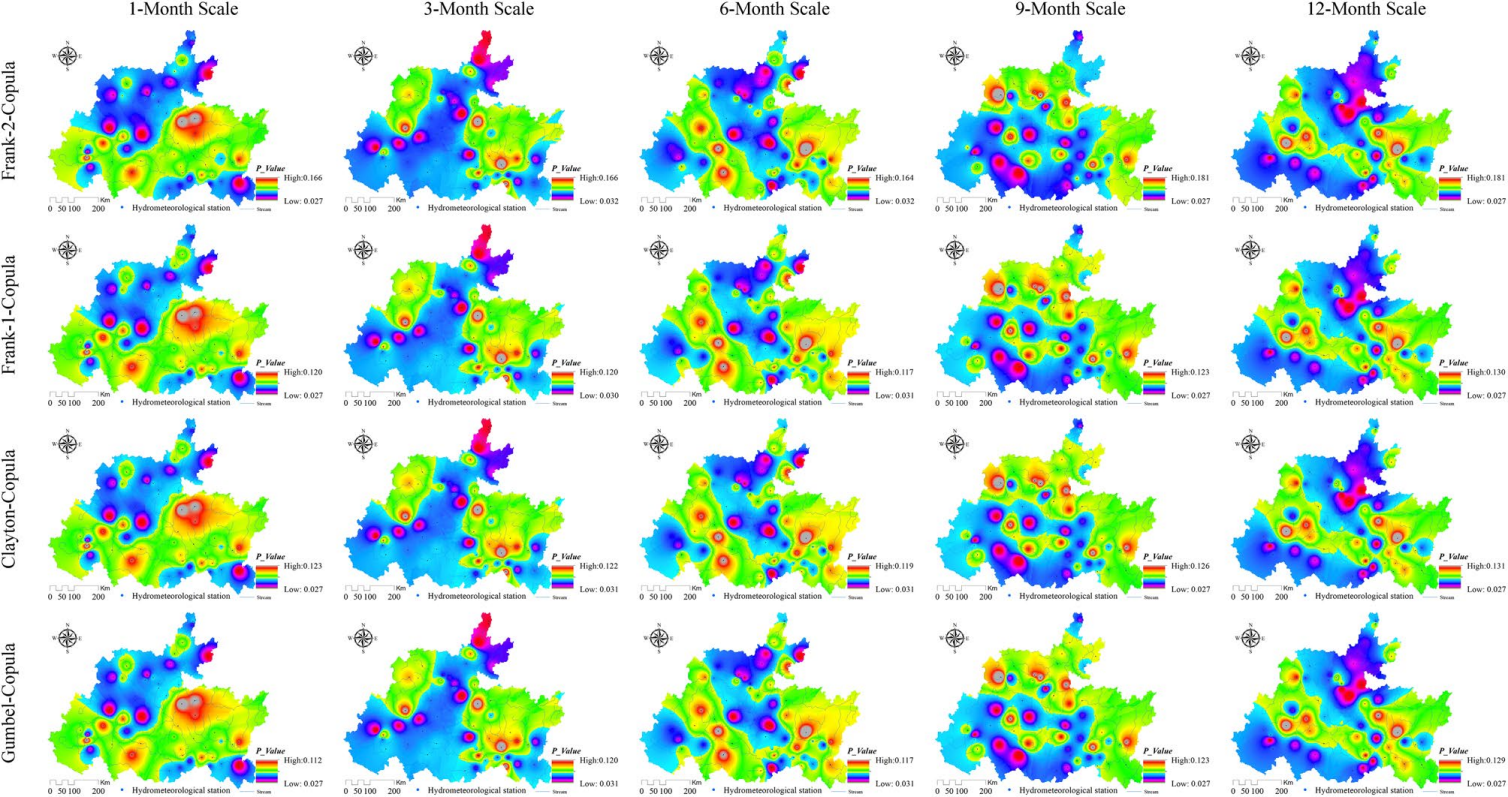
Spatial distribution characteristics of the copula joint probability between the lagged intensity and frequency simulated by the P-III distribution. Map was created with ArcGIS version 10.3 (https://www.arcgis.com/).

**Figure 13:**
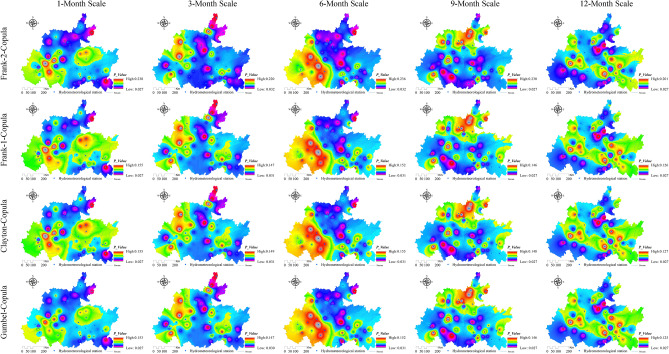
Spatial distribution characteristics of the copula joint probability between the lagged intensity and frequency simulated by the log-logistic distribution. Map was created with ArcGIS version 10.3 (https://www.arcgis.com/).

The watershed lagged intensity and frequency, whether in time or space, basically occur at the same time^9^. To better reveal the spatiotemporal evolution law of the watershed lagged effect, the joint probability between the lagged intensity and frequency was discussed using copula functions (such as the Gumbel copula, Clayton copula and Frank copula functions) in this paper (Figs. 10, 11, 12 and 13). In general, the spatial distributions of the Gumbel, Clayton, Frank-1 and Frank-2 copulas were relatively similar, with relatively small joint probabilities (Figs. 10, 11, 12 and 13). This result may have been due to the significant negative correlation (*R* < − 0.8, *Sig*. < 0.01) between the lagged intensity and frequency^22^. The influence of the time scale on the joint probability was also significant^54^. For instance, the joint probability simulated by the normal distribution at the 1-month scale gradually decreased from west to east, while the opposite trend was observed at the 9-month scale. The joint probability gradually increased along the north‒south direction at the 3-month scale and presented a spatial distribution along the northeast‒southwest direction at the 6- and 12-month scales. In particular, the joint probability at the 12-month scale displayed a significant decreasing trend from northwest to southeast (Fig. 10). The joint probability simulated by the log-normal distribution at the 3–9-month scales presented a spatial distribution with a significant north‒south trend. The overall joint probability at the 1-month scale was relatively high and presented the spatial pattern of strong–weak-strong alternation along the east‒west direction; at the 12-month scale, the join probability was relatively low and showed the spatial pattern of weak-strong–weak alternation along the east‒west direction (Fig. 11). In the simulation of the P-III distribution, the joint probability represented a decreasing trend from southeast to northwest at the 1-month scale, a declining trend from northeast to southwest at the 9-month scale, and a strong–weak alternation along the east‒west direction at the 3-month scale, as well as significant southeast‒northwest-direction distributions at the 6- and 12-month scales (Fig. 12). For the spatial distribution of the joint probability simulated by the log-logistic distribution, a significant decline from northeast to southwest was found at the 6-month scale, a southeast‒northwest-direction distribution was observed at the 12-month scale, a strong–weak alternation along the east‒west direction was presented at the 3-month scale, and weak-strong alternation along the south‒north direction was displayed at the 9-month scale (Fig. 13).

**Figure 14:**
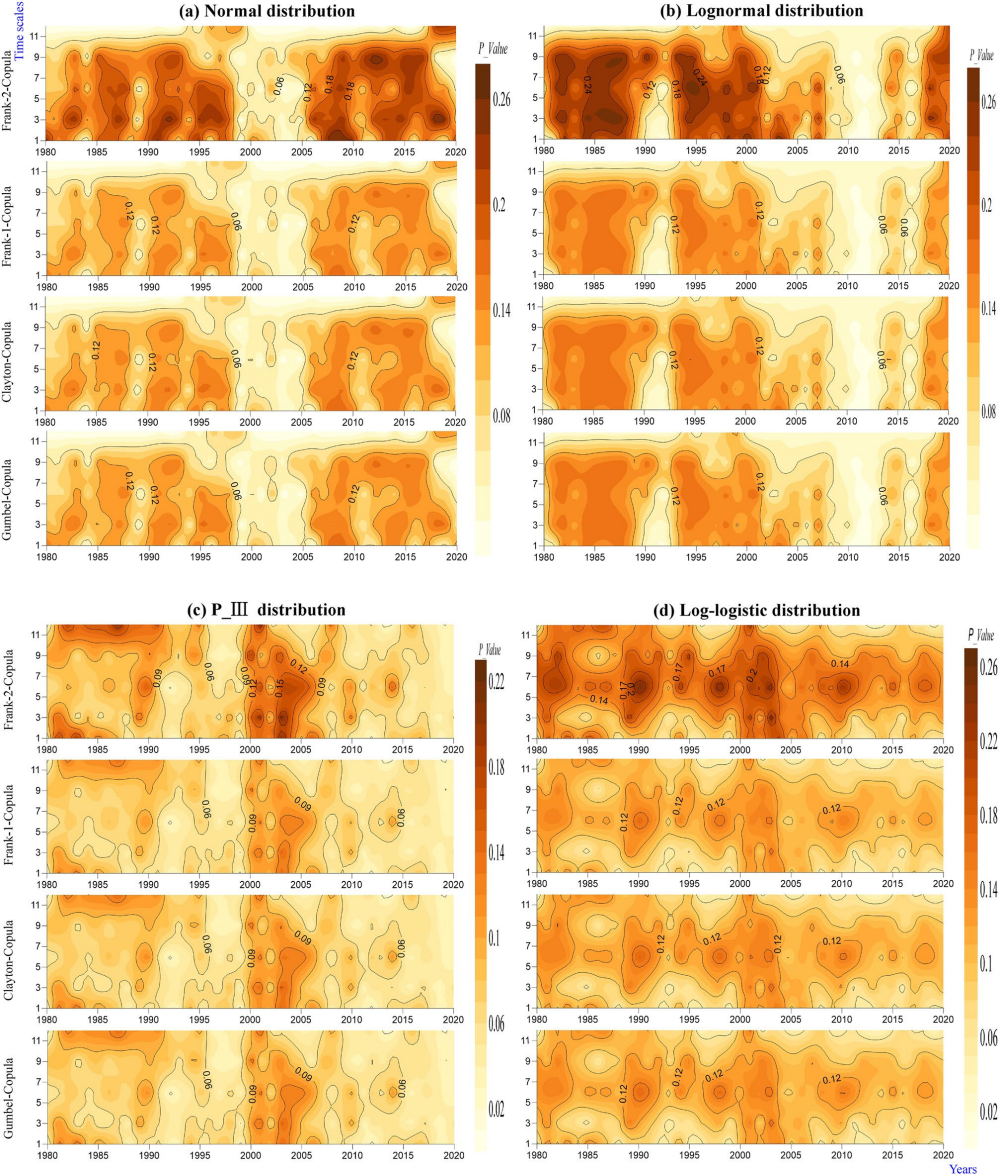
Temporal evolution characteristics of the copula joint probability between the lagged intensity and frequency simulated by the normal (**a**), log-normal (**b**), P-III (**c**) and log-logistic (**d**) distributions.

The joint probability periodically changed with increasing years, as shown in Fig. 14. The normal simulation results can be clearly divided into relatively strong probability periods from 1980 to 1999 and 2005 to 2020 and a relatively weak probability period from 1999 to 2005 (Fig. 14a). A strong probability period was found from 1980 to 2005 in the log-normal simulation results, a weak probability period was observed from 2005 to 2015, and a strengthening probability period was displayed from 2015 to 2020 (Fig. 14b). The joint probability simulated by the P-III distribution changed slightly from 1980 to 2000 and decreased periodically from 2000 to 2020 (Fig. 14c). Significant strong and weak periodic changes were presented in 1980 to 2020 in the log-logistic simulation results (Fig. 14d). With increasing time scales, the joint probabilities simulated by the normal and log-normal distributions gradually decreased (Fig. 14a,b, respectively). In the simulation results of the P-III distribution, the joint probability in 1980–2000 gradually increased from medium time scales (6 and 9 months) to short time scales (1 and 3 months) and long time scales (12 months) (Fig. 14b). However, just the opposite pattern was observed in the log-logistic simulation results; that is, the joint probability gradually weakened in the 1980 to 2020 period from medium time scales (6 and 9 months) to short time scales (1 and 3 months) and long time scales (12 months) (Fig. 14d).

## Discussion

### Theoretical analysis of the watershed lagged effect

Atmospheric precipitation flows into a river network after being intercepted by the vegetation layer and influenced by the soil layer and overland flow on the slope, then finally flows out at the outlet section of the basins after confluence through the river network^20,22,55^. Therefore, there is a certain lag time between rainfall and runoff, and all watersheds have a certain lagged effect in terms of this point of view^46^. Especially in areas with small surface relief and gentle slopes, the surface velocity of rainfall is slow, the infiltration rate is high, and the watershed lagged effect is particularly significant^17,25,56^. In regions far from the main river channel or located in the downstream regions of basins, the rainfall-runoff time is relatively long, the infiltration rate is high, and the basins show strong lag characteristics^57,58^. This study shows that the watershed lagged intensity presents spatial distribution patterns of strong in the east and south and weak in the west and north (Fig. 7). This result indicates a significant spatial distribution difference in watershed lagged effects. The distribution area of the severe lagged effect simulated by the log-normal distribution at the 12-month scale was as high as 92.12%, and those of the mild lagged effect at the 1- and 6-month scales were 93.09% and 82.04%, respectively. The distribution area of the moderate lagged level simulated by the P-III and log-logistic distributions ranged from 72.03 to 80.69% and from 50.65 to 84.36%, respectively (Fig. 4a). These results demonstrate that the watershed lagged effect has time scale effect characteristics^59,60^.

### Analysis of the characteristics of the watershed lagged effect

The watershed lagged effect is usually characterized by the lagged intensity and frequency, which are quantitative indicators used to evaluate the lagged effect^17,61^. These indicators are deeply affected by regional climate factors (e.g., the rainfall intensity, air temperature and evapotranspiration) and the basin media type and structure, as well as by the infiltration rate and lagged-flow volume of rainfall^17,61,62^. Some scholars have shown that in areas with high rainfall intensities and short rainfall durations, as well as high temperatures and strong evapotranspiration, the infiltration rates are low and the lagged-flow volumes are small, resulting in a nonsignificant watershed lagged effect. The watershed lagged effect is stronger the in summer and autumn seasons with relatively large rainfall and weaker in the spring and winter seasons with relatively small rainfall^2,63^. Vertically layered vegetation has a *reducing speed effect* on the vertical movement of rainfall; that is, it reduces the attack and damage impacts of rainfall on the watershed surface and enhances the infiltration of rainfall^9^. The horizontal distribution of vegetation has an *interception effect* on the lateral runoff of rainfall; that is, it slows the surface velocity of rainfall and enhances the infiltration of rainfall. Therefore, watershed vegetation coverage contributes to the watershed lagged effect to a certain extent^25,64^; in particular, the forest vegetation impact is particularly significant^30^, followed by the grassland and cultivated land impacts^2,3^. This study showed that lagged intensities corresponding to the mildly to severely lagged levels were found in the log-normal and P-III simulation results, while nonlagged to severely lagged levels were observed in log-logistic simulation results and the mildly lagged level was shown in the normal simulation results (Fig. 7). At the same time, the watershed lagged intensity and frequency occur simultaneously in both time and space with a smaller joint probability. This is due to the negative correlation (R < − 0.8, *Sig*. < 0.01) between the watershed lagged intensity and frequency^22^. The fitting effect of the joint probability by the Gumbel copula was the best according to the *AIC* and *BIC* test results, followed by the Clayton and Frank-1 copulas, while the Frank-2 copula was relatively weak. The watershed lagged effects obtained on different time scales had relatively large differences, and the *C*_*v*_ values of the nonlagged (except at the 6-month scale) and severely lagged (except at the 1-month scale) levels were greater than 1, while those of the mildly (except at the 12-month scale) and moderately lagged levels were less than 1 (Fig. 6, left). These results indicate that the watershed lagged effects on short time scales (1 and 3 months) are affected mainly by rainfall changes and are controlled by the water storage capacity on long time scales (12 months), while they are influenced by the combined effects of both rainfall variations and runoff regulation on medium time scales (6 and 9 months)^62,65^.

### Relationship between the watershed lagged effect and water storage

The watershed water storage plays a key role in the lagged-flow volume of the rainfall-runoff process^22,56,66^, especially in terms of the impact of basin elevation changes on the water storage capacity, which cannot be ignored^22,57^. Figure 1 shows that the basin elevation and river network density gradually decrease from west to east and/or from southwest to northeast, and the watershed lagged effect characteristics generally decline, especially the lagged frequency, the decline in which was particularly significant (Fig. 8). This finding indicates that the higher the basin elevation is, the deeper the erosion/dissolution base levels are buried; the higher the vertical distance from the basin surface to base level is, the thicker the basin thickness or the greater the watershed water-stored space is; and the stronger the watershed water storage capacity is, the more significant the watershed lagged effect is. The smaller the river network density is, the weaker the capacity of the catchment’s confluence is, and the longer the rainfall detention time is, the more significant the lag response of runoff to rainfall is^35,67^. Some previous studies have demonstrated that factors such as the basin relief and slope, as well as the drainage density and watershed shape, influence the ability of precipitation to flow into river networks and out of watersheds and are significantly related to low stream flow^68,69^. Several studies have confirmed the negative relationship between the drainage density and low flow^69^. It is possible that the negative relationship between low flow and the drainage density is partially due to the negative correlations between the drainage density and subsurface characteristics. The drainage density relates negatively to the base flow during droughts not only because it facilitates the removal of water in subsurface storage but also because it is a direct reflection of the subsurface storage conditions^70^. Simultaneously, soil is an important component factor of a basin, and soil particles have a strong adsorption effect on atmospheric precipitation. Soil space is the smallest storage unit or sink of atmospheric precipitation and comprehensively reflects the watershed water storage capacity^21^. The basin lithology, especially the karst lithology, not only comprises many types and impure textures but also suggests an alternative distribution of different lithological types and forms different lithological combination structures. These different structural characteristics, such as karst gaps, pores and pipelines, as well as underground karst caves developed under the karst effect, provide space for rainfall detained on the surface and underground in watersheds^36^. The lithology of karst basins compared to nonkarst basins has a stronger water storage capacity, resulting in the lagged effect in karst basins being relatively significant. Especially at relatively long time scales, the watershed lagged effect is the result of the combined effect of both regional climate change and watershed water storage^71^. With the development of the social economy, human demands for water resources have undergone significant changes, leading to a significant impact of human activities on watershed lagged effects^3,7,45^. This study shows that watershed lagged intensity simulated by the P-III and log-logistic distributions in different years presented a significant strong–weak alternation, and in the normal distribution simulation, the watershed lagged intensity gradually increased with an increasing time scale (Fig. 9). The lagged frequency simulated by the normal distribution was enhanced from 1980–2000 and 2006–2020 and weakened from 2000 to 2006 (Fig. 9).

### Limitations of this study

However, due to data limitations, this study selected only rainfall-runoff data to calculate the lagged-flow volume by a distributed lag regression model and discuss the characteristics of the watershed lagged effect. In further research, we will focus on the coupling impacts of different karst basin of different spatial scales and different hydrometeorological data time scales on the watershed lagged effects. In particular, the uncertain influences of climate change on the time series of the lagged-flow volume and the destruction and reconstruction of human activities on the underlying surfaces of basins are expected to influence the runoff generation and confluence mechanisms in karst basins. Comprehensively considering meteorological factors, basin underlying surfaces and human activity factors, we will simulate the rainfall-runoff process using the SWAT model and GCMs^72^, build a response index of runoff to rainfall, and further reveal the mechanism of the lagged effect in karst drainage basins.

## Conclusion

Compared to normal basins, karst basins contain soluble aqueous media in which spaces of different sizes develop under differential erosion or the dissolution of soluble water. These spaces provide places for the retention of rainfall on the surface and underground and result in the strong lagged response of runoff to rainfall. According to the smallest criterion of *MSEs*, the log-normal distribution is the optimal model for simulating the lagged effect in karst basins, and the fitting effects of other models were relatively weak. Affected by the spatial distribution of and seasonal variation in rainfall, the watershed lagged effect has significant time-scale-dependent characteristics, especially the coefficient of variation (*C*_*v*_) of lagged intensities, which is greater than 1 on the 1-, 3- and 12-month scales and less than 1 on the 6- and 9-month scales. These results indicate that the lagged effect in karst basins is influenced mainly by rainfall changes on short time scales (1 and 3 months) and is controlled by both rainfall variations and runoff regulation on long time scales (12 months). The watershed lagged effect is usually characterized by a lagged intensity and frequency. Among them, the lagged frequencies simulated by the log-normal, P-III and log-logistic distributions in karst basins are relatively high, while that simulated by the normal distribution is relatively low. In particular, medium, medium–high and high frequencies are found in the log-normal and P-III simulation results, while medium–low and low frequencies are shown in the normal simulation results. However, the joint probability between the lagged intensity and frequency is generally low, possibly because there is a significant negative correlation between these factors (*R* < − 0.8, *Sig*. < 0.01), and the water storage capacity in karst basins shows spatial distribution differences. For the simulation of joint probabilities, the Gumbel copula fits best, followed by the Clayton and Frank-1 copulas, while the Frank-2 copula is relatively weak. Simultaneously, the rainfall-runoff process is influenced by the destruction and reconstruction of human activities on watershed media, resulting in both the lagged intensity and frequency having significant chronological differences. Therefore, the watershed lagged effect is the combined result of climate change, the basin media and structure, and human activities. This study confirms that rainfall deficits are a necessary but insufficient condition for basin drought occurrence, especially in karst basins. The watershed lagged effect determines and/or controls the propagation process from meteorological drought to agricultural or hydrological drought as well as the conversion mechanisms between different drought types.

## Data Availability

All data generated or analyzed during this study are included in this published article.
